# Response to Biologic Therapy in Skin of Colour Participants With Moderate-to-Severe Psoriasis and Atopic Dermatitis: A Systematic Review

**DOI:** 10.1177/12034754241260023

**Published:** 2024-06-07

**Authors:** Hibo Rijal, Naïla Bouadi, Abrahim Abduelmula, Khalad Maliyar, Adrienn N. Bourkas, Jorge-Ryan Georgakopoulos, Jensen Yeung

**Affiliations:** 1 Queen’s University School of Medicine, Kingston, ON, Canada; 2 Faculty of Medicine, Université Laval, Québec City, QC, Canada; 3 Division of Dermatology, Department of Medicine, University of Toronto, Toronto, ON, Canada; 4 Women’s College Research Institute, Women’s College Hospital, Toronto, ON, Canada; 5 Sunnybrook Health Sciences Centre, Toronto, ON, Canada

**Keywords:** atopic eczema, psoriasis, equity/disparity, clinical trial, patients, outcome measurement

## Abstract

There has been a call to action to enhance representation of non-white individuals in dermatology clinical trials. Investigations in differential response to treatment across populations are limited, particularly in conditions of commonality, impact, distinct presentation, and diagnosis in non-white participants, such as atopic dermatitis and psoriasis. This systematic review summarized and identified if biologic treatment outcomes in moderate-to-severe atopic dermatitis and psoriasis varied in skin of colour (SOC) participants in phase 3 trials. MEDLINE, COCHRANE, and EMBASE databases were used to conduct the search following PROSPERO registration. Following screening of 3209 articles, 11 studies were collected with 1781 SOC participants with a mean age of 40.99 ± 6.3 years (range: 30.6-51.6 years). Male participants accounted for 76.9% (n = 1370/1781) of the sample, and Chinese, Japanese, Taiwanese, and Korean participants accounted for 64.3%, 24.2%, 4.5%, and 3.4% of participants, respectively. Participants with atopic dermatitis were treated with dupilumab (n = 216/388) and participants with psoriasis were treated with adalimumab (n = 313/1393), bimekizumab (n = 62/1393), ixekizumab (n = 13/1393), secukinumab (n = 117/1393), and ustekinumab (n = 289/1393). No significant SOC population-based outcomes were found across treatment groups. However, differences in baseline characteristics or comorbidities were found, suggesting race or ethnic background should be considered when treatment is prescribed in psoriasis or atopic dermatitis. Although no significant SOC participant differential response to treatment were found, large-scale randomized controlled trials investigating comparable treatment outcomes and stratifying results by SOC population in atopic dermatitis and psoriasis are warranted to confirm these findings.

## Introduction

The realm of dermatology clinical trials reveals marked imbalances in the diversity of participants enlisted. A study conducted by Chen et al, delved into the evolution of race and ethnicity reporting within dermatology randomized controlled trials both prior to and after 2015.^1^ The findings indicated an augmentation in the reporting of racial and ethnic data after 2015. However, despite this increase, the proportion of non-white participants displayed no significant change, remaining stagnant.^1^ While contemporary efforts are underway to enhance the comprehensiveness and inclusivity of clinical trials in the United States, the extent to which these initiatives have facilitated advancements in research pertaining to skin of colour remains unclear.^2^ The stratification of data according to race or ethnicity stands as a crucial endeavour, to support insights into the broader applicability of study outcomes. This facet assumes particular significance within the dermatological domain, where conditions manifesting in individuals with diverse skin tones can pose intricate diagnostic challenges, leading to disparate treatment ramifications.^3^

Atopic dermatitis and psoriasis reflect these differences. Atopic dermatitis is a common chronic inflammatory skin disease usually beginning in infancy. It presents with dry skin, pruritis, lichenification, and eczematous lesions.^4^ Notably, in patients with skin of colour, atopic dermatitis assumes distinct nuances, marked by dyspigmentation and a pronounced predilection for extensor involvement, in comparison to their Caucasian counterparts.^5^ The incidence of atopic dermatitis further underscores these ethnic differentials. Asian and Black pediatric cohorts exhibit heightened tendencies to seek medical attention for their symptoms and consequent diagnostic evaluation of atopic dermatitis, in comparison to their Caucasian peers.^6,7^ Similarly, psoriasis is a chronic inflammatory disease with autoimmune pathogenic characteristics^8^ that vary based on skin of colour. Psoriasis is characterized by clearly demarcated erythematous, plaques, with thick, silvery scale.^8^ In non-white individuals, psoriatic plaques often present with dyspigmentation, and the erythematous component can be subtly appreciated, assuming shades of deep brown, be violaceous, or as a light pink, which is a departure from the pink-to-red hues commonly observed in individuals with lighter skin.^8^ Psoriasis has geographic variability as it occurs in 11% of Scandinavian and Caucasian populations and has lower prevalence in Asian and African populations.^9^ Despite the lower incidence of psoriasis in patients with skin of colour, disconcerting trends have emerged such as delayed diagnosis in individuals with darker skin compared to their lighter-skinned counterparts.^10^ These observations collectively underscore the interplay between ethnicity and the incidence, clinical expression and diagnosis of atopic dermatitis and psoriasis, propelling the need for further investigation in diverse population subsets.

The rise of biologic therapies has revolutionized treatment for cutaneous and systemic diseases such as atopic dermatitis and psoriasis by targeting specific molecules within their molecular pathways.^11^ However, few studies investigate if differences in biologic treatment response exist based on racial or ethnic diversity.^12,13^ In this systematic review, we summarize moderate-to-severe atopic dermatitis and psoriasis biologic treatment outcomes in skin of colour populations in phase 3 trials. This review will provide valuable insight into the different biologic treatment outcomes in these patient populations for dermatologists.

## Methods

This systematic review was conducted under the accordance of the Preferred Reporting Items for Systematic Reviews and Meta-Analyses (PRISMA) guidelines (Supplemental Figure 1) and registered on Prospero (ID: CRD42023407319). Searches on COCHRANE, MEDLINE, and EMBASE databases were conducted in OVID on February 14, 2023. The following keywords and variations were searched: “atopic dermatitis” and “psoriasis.” Searches were restricted to after 2008 and the English language. Studies were included if they (1) conducted a phase 3 trial, (2) included adult participants, (3) documented participants with moderate to severe atopic dermatitis or psoriasis, (4) used biologics as an intervention, and (5) documented separate results and outcomes of treatment in non-white participants (Supplemental Figure 2). Two reviewers (HR and NB) independently conducted title and abstract screening and full-text review in accordance with eligibility criteria. Conflicts were resolved by a third reviewer (AA) in both stages of screening. Additional relevant studies were included following review of reference list of eligible articles. Two reviewers (HR and ANB) completed risk of bias assessment. All studies were independently reviewed. Version 2 of the Cochrane Risk of Bias (ROB) tool was used to assess full risk of bias in the included randomized controlled trials (Supplemental Figure 3). The Cochrane ROB2 tool identifies risk of bias in study trial design, reporting and conduct to evaluate quality effectiveness of the studies included.^14^ A descriptive analysis was completed to account for heterogeneity of the included studies. The Oxford Centre for Evidence-Based Medicine 2011 Levels of Evidence was used by 2 reviewers (HR and NB) to report quality of evidence (Supplemental Table 1).^15^

Two reviewers (HR and NB) extracted data from each eligible study. Country of publication, patient demographic characteristics, intervention, and treatment outcomes were extracted and documented (Supplemental Table 1). Extraction and analysis of the following posttreatment outcomes were conducted:

1. Investigator Global Assessment (IGA): defined as IGA 0/1
2. Psoriasis Area and Severity Index (PASI): defined as PASI 100/90/75/50
3. Eczema Area and Severity Index (EASI): defined as EASI 100/90/75/50
4. Dermatology Life Quality Index (DLQI): defined as DLQI 0/1
5. Body Surface Area (BSA): defined as % change from baseline
6. Reported adverse events

## Results

After duplicates were removed, the search retrieved 3209 articles. From this search, 612 articles were selected for full-text review following title and abstract screening. Eleven articles were selected for data extraction that met the review’s eligibility criteria (Supplemental Figure 2). Data extraction of the 11 studies yielded analysis for 1781 participants. Male participants represented 76.9% of study participants (n = 1370/1781). At presentation, participants had a mean age of 40.99 ± 6.3 years (range of 30.6-51.6 years; Supplemental Table 1).

Studies were conducted at centres in China, Japan, Korea, Taiwan, the United States, Netherlands, Australia, Germany, Canada, and the United Kingdom (Supplemental Figure 4). A total of 62.7% (n = 1116), 24.2% (n = 431), 8.0% (n = 143), 4.4% (n = 79), 0.2% (n = 4), 0.1% (n = 2), 0.1% (n = 2), 0.1% (n = 2), 0.1% (n = 1), and 0.1% (n = 1) of participants were recruited from centres in China, Japan, Taiwan, Korea, Canada, Netherlands, Australia, Germany, the United States, and the United Kingdom, respectively (Supplemental Figure 5). A total of 64.3% (n = 1146), 24.2% (n = 431), 4.5% (n = 81), and 3.4% of participants (n = 61) participants were categorized as ethnically Chinese, Japanese, Taiwanese, and Korean, respectively (Supplemental Figure 6). A total of 3.5% (n = 62) participant’s specific ethnic background were not recorded (Supplemental Figure 6).

Of the 1781 participants with moderate-to-severe psoriasis and atopic dermatitis, 388 had atopic dermatitis and 1393 had psoriasis. Among those with atopic dermatitis, 55.7% (n = 216) were treated with dupilumab. Among those with psoriasis, 22.5% (n = 313), 4.5% (n = 62), 0.9% (n = 13), 8.4% (n = 117), and 20.7% (n = 289) were treated with adalimumab, bimekizumab, ixekizumab, secukinumab, and ustekinumab, respectively (Supplemental Table 1).

Treatment periods ranged from 12 to 244 weeks. Concomitant medications were reported in 30.2% (n = 117) of participants with atopic dermatitis, of which 100% were topical corticosteroids. No concomitant interventions were reported in psoriasis participants (Supplemental Table 1).

### Biologic Treatment Response in Psoriasis

By week 16, responses in PASI 100/90/75 were reported highest in skin of colour participant cohorts treated with bimekizumab, reporting levels of 52.6% (n = 32/62), 85.5% (n = 53/62), and 95.2% (n = 59/62), respectively. PASI 50 was reported the highest in ustekinumab-treated participants in comparison to other biologics treating skin of colour psoriasis in this review, with 94.4% (n = 151/160) of the cohort reaching PASI 50 by week 16. In IGA 0/1 and DLQI of 0/1, by week 16, highest responses were in participants treated with bimekizumab with 82.3% (n = 51/62) and 82.3% (n = 51/62) reported, respectively. There were 1074 adverse events reported in participants with psoriasis. Infections, specifically nasopharyngitis, was the most frequently reported adverse event, highest in treatment involving Chinese participants with ustekinumab (n = 26) versus placebo (n = 28). There were 39 discontinuations and no deaths reported due to adverse events in participants with psoriasis (Supplemental Table 1).

### Biologic Treatment Response in Atopic Dermatitis

All participants with atopic dermatitis were treated with dupilumab (n = 216), of which 40.2% (n = 33/82), 57.3% (n = 47/82), and 70.7% (n = 58/82) reached respective levels of EASI 90/75/50 by week 16. Highest reported IGA 0/1 was documented in 31.2% (n = 15/47) of participants, and a BSA % change from baseline (CFB) of −37.8 ± 2.13 (n = 82) by week 16. In participants with atopic dermatitis, there were 190 reported adverse events, most frequently skin and subcutaneous tissue disorders in Chinese participants treated with dupilumab (n = 40) versus placebo (n = 26). There were 11 trial discontinuations and no deaths reported due to adverse events in participants with atopic dermatitis (Supplemental Table 1).

## Discussion

From this review of phase 3 trials, it was identified that the most common biologic used to investigate outcomes of moderate-to-severe psoriasis in skin of colour populations was adalimumab (n = 313/1781).^16,17^ In Chinese participants, less individuals treated with adalimumab (93%; n = 121) compared to adalimumab biosimilar HLXO3 (96%; n = 126) reached PASI 75.^15^ Comparatively, in studies that investigated adalimumab to placebos in Chinese patients, patients experienced a greater proportion of PASI 75 response in the biologic group^18^ suggesting efficacy of adalimumab biosimilars in inhibiting the TNF-α pathway in Chinese patients with psoriasis.^19^

All participants treated with bimekizumab were of Japanese origin. Those with moderate-to-severe psoriasis treated with bimekizumab experienced a greater proportion of achieved treatment outcomes (PASI 100/90/75, IGA 0/1, DLQI 0/1) than those of the placebo group.^20^ This is in accordance with the larger BE VIVID study demonstrating greater proportion of achieved treatment outcomes in bimekizumab-treated participants compared to ustekinumab or placebo.^21^ Positive treatment responses (mean PASI and DLQI) were also reported in Japanese participants treated with ixekizumab.^22^

In this review, 8.4% (n = 117) of participants of psoriasis were treated with secukinumab. Of these groups, 58, 36, and 23 participants were categorized as Japanese, Taiwanese, and non-specified Asian, respectively.^23-25^ Japanese and Taiwanese participants reported favourable treatment outcomes of IGA0/1, PASI 100/90/75, and DLQI 0/1 in comparison against placebo. Non-specified Asian participants similarly experienced improved IGA0/1, PASI 100/90/75, and DLQI 0/1 scores when treated with secukinumab in comparison to ustekinumab.^23-25^ These findings support previous studies including Asian participants, that reported favourable treatment responses to secukinumab in treating their moderate-to-severe psoriasis.^1,26,27^

Furthermore, 20.7% (n = 289) of patients with psoriasis underwent ustekinumab treatment, spanning Korean (n = 31),^28^ Taiwanese (n = 30),^28^ Chinese (n = 160),^29^ Japanese (n = 29),^20^ and non-specified Asian participants (n = 39).^25^ Notably, Korean, Taiwanese, and Chinese participants displayed improved treatment outcomes (PASI 100/90/75) with ustekinumab over placebo^28,29^ consistent with prior East Asian-specific responses.^1,30,31^ Japanese patients showed greater treatment outcomes with ustekinumab than placebo,^20^ but lesser when compared to bimekizumab, which is consistent with previous findings comparing the 2 treatment responses in Japanese patients.^21^ These insights provide a concise depiction of distinct East Asian population responses to ustekinumab and bimekizumab, enhancing our therapeutic understanding.

Among the cohort of 216 atopic dermatitis participants subjected to dupilumab treatment at study commencement, discernible trends emerge. Chinese (n = 82) and Japanese participants (n = 134) exhibited heightened attainment of treatment objectives, as indicated by parameters encompassing EASI 90/75/50, IGA 0/1, and BSA % CFB when juxtaposed against participants treated with placebo.^32,33^ These findings resonate with trial data specific to these populations, consistently underscoring similar treatment responses and mitigated adverse events.^8,34^

This systematic review shows that biologics are safe and efficacious to use when treating skin of colour patients with moderate-to-severe psoriasis or atopic dermatitis. These findings substantiate the viability of targeting signalling pathways implicated in curbing the release of pro-inflammatory cytokines, specifically tumor necrosis factor-alpha (TNF-α) and interleukins 17, 4R, 12 and 23 as potent therapeutic strategies for mitigating moderate-to-severe atopic dermatitis and psoriasis in these distinct demographic subsets.^17,35-37^ Among 11 studies, 5 featured subanalyses comparing non-white subsets to the global study population.^19,23-25,32^ Notably, Taiwanese participants displayed enhanced psoriasis response to secukinumab compared to Japanese and global cohorts.^24^ While not statistically significant, this trend is noteworthy. Skin of colour-specific outcomes were largely consistent across studies.^28,29,33^

Several limitations characterize this systematic review. Notable among these is the relatively small sample size, which subsequently undermines the broad generalizability of the findings. Furthermore, this review comprised of pre-dominantly East Asian participants and no other ethnicities, thereby constricting the breadth of data for analysis. The limitation of participants to those of East Asian origin, reflective of location and population in which trials were conducted, further affects generalizability of biologic therapy response findings to other skin of colour demographics. Studies including individual response to treatment did not account for individual genetic or baseline variability potentially confounding results. Furthermore, not all studies stratified by intervention reported the same treatment outcomes of IGA, PASI, EASI, DLQI, or BSA or same timelines, limiting comparability across skin of colour groups.

Ongoing efforts are directed toward increasing the representation of non-white participants in dermatology trials.^38^ Notably, there are a lack of reviews that stratify skin of colour responses in phase 3 trials involving individuals with moderate-to-severe atopic dermatitis or psoriasis. Our investigation reveals that significant differences in therapeutic responses to biologics among skin of colour patients were not identified. Larger sample sizes must be used in future investigations to confirm the finding of this review.

## Supplemental Material

sj-docx-1-cms-10.1177_12034754241260023 – Supplemental material for Response to Biologic Therapy in Skin of Colour Participants With Moderate-to-Severe Psoriasis and Atopic Dermatitis: A Systematic Review Supplemental material, sj-docx-1-cms-10.1177_12034754241260023 for Response to Biologic Therapy in Skin of Colour Participants With Moderate-to-Severe Psoriasis and Atopic Dermatitis: A Systematic Review by Hibo Rijal, Naïla Bouadi, Abrahim Abduelmula, Khalad Maliyar, Adrienn N. Bourkas, Jorge-Ryan Georgakopoulos and Jensen Yeung in Journal of Cutaneous Medicine and Surgery

sj-pdf-2-cms-10.1177_12034754241260023 – Supplemental material for Response to Biologic Therapy in Skin of Colour Participants With Moderate-to-Severe Psoriasis and Atopic Dermatitis: A Systematic Review Supplemental material, sj-pdf-2-cms-10.1177_12034754241260023 for Response to Biologic Therapy in Skin of Colour Participants With Moderate-to-Severe Psoriasis and Atopic Dermatitis: A Systematic Review by Hibo Rijal, Naïla Bouadi, Abrahim Abduelmula, Khalad Maliyar, Adrienn N. Bourkas, Jorge-Ryan Georgakopoulos and Jensen Yeung in Journal of Cutaneous Medicine and Surgery

sj-pdf-3-cms-10.1177_12034754241260023 – Supplemental material for Response to Biologic Therapy in Skin of Colour Participants With Moderate-to-Severe Psoriasis and Atopic Dermatitis: A Systematic Review Supplemental material, sj-pdf-3-cms-10.1177_12034754241260023 for Response to Biologic Therapy in Skin of Colour Participants With Moderate-to-Severe Psoriasis and Atopic Dermatitis: A Systematic Review by Hibo Rijal, Naïla Bouadi, Abrahim Abduelmula, Khalad Maliyar, Adrienn N. Bourkas, Jorge-Ryan Georgakopoulos and Jensen Yeung in Journal of Cutaneous Medicine and Surgery

sj-pdf-4-cms-10.1177_12034754241260023 – Supplemental material for Response to Biologic Therapy in Skin of Colour Participants With Moderate-to-Severe Psoriasis and Atopic Dermatitis: A Systematic Review Supplemental material, sj-pdf-4-cms-10.1177_12034754241260023 for Response to Biologic Therapy in Skin of Colour Participants With Moderate-to-Severe Psoriasis and Atopic Dermatitis: A Systematic Review by Hibo Rijal, Naïla Bouadi, Abrahim Abduelmula, Khalad Maliyar, Adrienn N. Bourkas, Jorge-Ryan Georgakopoulos and Jensen Yeung in Journal of Cutaneous Medicine and Surgery

sj-pdf-5-cms-10.1177_12034754241260023 – Supplemental material for Response to Biologic Therapy in Skin of Colour Participants With Moderate-to-Severe Psoriasis and Atopic Dermatitis: A Systematic Review Supplemental material, sj-pdf-5-cms-10.1177_12034754241260023 for Response to Biologic Therapy in Skin of Colour Participants With Moderate-to-Severe Psoriasis and Atopic Dermatitis: A Systematic Review by Hibo Rijal, Naïla Bouadi, Abrahim Abduelmula, Khalad Maliyar, Adrienn N. Bourkas, Jorge-Ryan Georgakopoulos and Jensen Yeung in Journal of Cutaneous Medicine and Surgery

sj-pdf-6-cms-10.1177_12034754241260023 – Supplemental material for Response to Biologic Therapy in Skin of Colour Participants With Moderate-to-Severe Psoriasis and Atopic Dermatitis: A Systematic Review Supplemental material, sj-pdf-6-cms-10.1177_12034754241260023 for Response to Biologic Therapy in Skin of Colour Participants With Moderate-to-Severe Psoriasis and Atopic Dermatitis: A Systematic Review by Hibo Rijal, Naïla Bouadi, Abrahim Abduelmula, Khalad Maliyar, Adrienn N. Bourkas, Jorge-Ryan Georgakopoulos and Jensen Yeung in Journal of Cutaneous Medicine and Surgery

sj-pdf-7-cms-10.1177_12034754241260023 – Supplemental material for Response to Biologic Therapy in Skin of Colour Participants With Moderate-to-Severe Psoriasis and Atopic Dermatitis: A Systematic Review Supplemental material, sj-pdf-7-cms-10.1177_12034754241260023 for Response to Biologic Therapy in Skin of Colour Participants With Moderate-to-Severe Psoriasis and Atopic Dermatitis: A Systematic Review by Hibo Rijal, Naïla Bouadi, Abrahim Abduelmula, Khalad Maliyar, Adrienn N. Bourkas, Jorge-Ryan Georgakopoulos and Jensen Yeung in Journal of Cutaneous Medicine and Surgery

## References

[1] Chen YC Huang YT Yang CC , et al Real-world efficacy of biological agents in moderate-to-severe plaque psoriasis: an analysis of 75 patients in Taiwan. PLoS One. 2020;15(12):e0244620. doi:10.1371/journal.pone.0244620 PMC7771674 33373425

[2] US Department of Health and Human Services. Guidance for industry: Collection of race and ethnicity data in clinical trials. 2016. Accessed August 27, 2015. http://www.fda.gov/downloads/regulatoryinformation/guidances/ucm126396.pdf

[3] Alexis AF Blackcloud P. Psoriasis in skin of color: epidemiology, genetics, clinical presentation, and treatment nuances. J Clin Aesthet Dermatol. 2014;7(11):16-24. PMC4255694 25489378

[4] Kolb L Ferrer-Bruker SJ. Atopic Dermatitis. In: StatPearls. StatPearls Publishing; 2022. 28846349

[5] Adawi W Cornman H Kambala A Henry S Kwatra SG. Diagnosing atopic dermatitis in skin of color. Dermatol Clin. 2023;41(3):417-429. doi:10.1016/j.det.2023.02.003 37236711

[6] Horii KA Simon SD Liu DY Sharma V. Atopic dermatitis in children in the United States, 1997-2004: visit trends, patient and provider characteristics, and prescribing patterns. Pediatrics. 2007;120:e527-e534. 10.1542/peds.2007-0289 17766497

[7] Janumpally SR Feldman SR Gupta AK Fleischer AB Jr. In the United States, blacks and Asian/Pacific Islanders are more likely than whites to seek medical care for atopic dermatitis. Arch Dermatol. 2002;138(5):634-637. 12020225 10.1001/archderm.138.5.634

[8] Igawa K Fujita H Tajima Y , et al Reduction in hospitalisations with dupilumab in Japanese adults with atopic dermatitis in a real-world setting. JEADV Clin Pract. 2023;2:543-548. doi:10.1002/jvc2.151

[9] Rendon A Schäkel K. Psoriasis pathogenesis and treatment. Int J Mol Sci. 2019;20(6):1475. doi:10.3390/ijms20061475 30909615 PMC6471628

[10] Khanna R Khanna R Desai SR. Diagnosing psoriasis in skin of color patients. Dermatol Clin. 2023;41(3):431-434. doi:10.1016/j.det.2023.02.002 37236712

[11] Hassan I Aleem S Sheikh G Anwar P. Biologics in dermatology: a brief review. Br J Med Pract. 2013;6(4):a629.

[12] Enos CW Yi JZ McLean RR , et al Similar response to biologic therapy across racial and ethnic groups among patients with psoriasis enrolled in the CorEvitas Psoriasis Registry. J Am Acad Dermatol. 2022;87(5):1087-1089. doi:10.1016/j.jaad.2022.05.038 35643239

[13] Ferguson JE Seger EW White J McMichael A. Racial/ethnic differences in treatment efficacy and safety for moderate-to-severe plaque psoriasis: a systematic review. Arch Dermatol Res. 2023;315(1):41-50. doi:10.1007/s00403-022-02324-4 35050396

[14] Sterne JAC Savović J Page MJ , et al RoB 2: a revised tool for assessing risk of bias in randomised trials. BMJ. 2019;366:l4898. doi:10.1136/bmj.l4898 31462531

[15] Oxford Centre for Evidence-Based Medicine. Levels of evidence (March 2009). 2016. Accessed July 15, 2023. https://www.cebm.net/2009/06/oxford-centre-evidence-based-medicine-levels-evidence-march

[16] Cai L Li L Cheng H , et al Efficacy and safety of HLX03, an adalimumab biosimilar, in patients with moderate-to-severe plaque psoriasis: a randomized, double-blind, phase III study. Adv Ther. 2022;39(1):583-597. doi10.1007/s12325-021-01899-0 34816373 10.1007/s12325-021-01899-0 PMC8799567

[17] Yu C Zhang F Ding Y , et al A randomized, double-blind phase III study to demonstrate the clinical similarity of biosimilar SCT630 to reference adalimumab in Chinese patients with moderate to severe plaque psoriasis. Int Immunopharmacol. 2022;112:109248. doi:10.1016/j.intimp.2022.109248 36126411

[18] Cai L Gu J Zheng J , et al Efficacy and safety of adalimumab in Chinese patients with moderate-to-severe plaque psoriasis: results from a phase 3, randomized, placebo-controlled, double-blind study. J Eur Acad Dermatol Venereol. 2017;31(1):89-95. doi:10.1111/jdv.13746 27504914 PMC5215651

[19] Gerriets V Goyal A Khaddour K. Tumor Necrosis Factor Inhibitors. In: StatPearls. StatPearls Publishing; 2022. 29494032

[20] Asahina A Okubo Y Morita A , et al Bimekizumab efficacy and safety in japanese patients with plaque psoriasis in BE VIVID: a phase 3, ustekinumab and placebo-controlled study. Dermatol Ther. 2023;13(3):751-768. doi:10.1007/s13555-022-00883-y PMC9984664 36648594

[21] Reich K Papp KA Blauvelt A , et al Bimekizumab versus ustekinumab for the treatment of moderate to severe plaque psoriasis (BE VIVID): efficacy and safety from a 52-week, multicentre, double-blind, active comparator and placebo controlled phase 3 trial. Lancet. 2021;397(10273):487-498. doi:10.1016/S0140-6736(21)00125-2 33549193

[22] Okubo Y Mabuchi T Iwatsuki K , et al Long-term efficacy and safety of ixekizumab in Japanese patients with erythrodermic or generalized pustular psoriasis: subgroup analyses of an open-label, phase 3 study (UNCOVER-J). J Eur Acad Dermatol Venereol. 2019;33(2):325-332. doi:10.1111/jdv.15287 30317671 PMC6587497

[23] Ohtsuki M Morita A Abe M , et al; ERASURE Study Japanese Subgroup. Secukinumab efficacy and safety in Japanese patients with moderate-to-severe plaque psoriasis: subanalysis from ERASURE, a randomized, placebo-controlled, phase 3 study. J Dermatol. 2014;41(12):1039-1046. doi:10.1111/1346-8138.12668 25354738

[bibr24-12034754241260023] Wu NL Hsu CJ Sun FJ Tsai TF. Efficacy and safety of secukinumab in Taiwanese patients with moderate to severe plaque psoriasis: subanalysis from ERASURE phase III study. J Dermatol. 2017;44(10):1129-1137. doi:10.1111/1346-8138.13900 28493369

[25] Lee MG Huang YH Lee JH , et al Secukinumab demonstrates superior efficacy and a faster response in clearing skin in Asian subjects with moderate to severe plaque psoriasis compared with ustekinumab: subgroup analysis from the CLEAR study. J Dermatol. 2019;46(9):752-758. doi:10.1111/1346-8138.15004 31342560

[26] Fujita H Ohtsuki M Morita A , et al Safety and effectiveness of secukinumab in psoriasis vulgaris and psoriatic arthritis: real-world evidence in Japan. J Dermatol. 2021;48(2):175-183. doi:10.1111/1346-8138.15655 33099791 PMC7894540

[27] Hsieh CY Tsai TF. Effectiveness and safety of reduced-dose secukinumab switching to risankizumab for psoriasis patients in Asian population: a case series. Exp Dermatol. 2023;32(7):1048-1050. doi:10.1111/exd.14810 37032610

[28] Tsai TF Ho JC Song M , et al; PEARL Investigators. Efficacy and safety of ustekinumab for the treatment of moderate-to-severe psoriasis: a phase III, randomized, placebo-controlled trial in Taiwanese and Korean patients (PEARL). J Dermatol Sci. 2011;63(3):154-163. doi:10.1016/j.jdermsci.2011.05.005 21741220

[29] Zhu X Zheng M Song M , et al; LOTUS Investigators. Efficacy and safety of ustekinumab in Chinese patients with moderate to severe plaque-type psoriasis: results from a phase 3 clinical trial (LOTUS). J Drugs Dermatol. 2013;12(2):166-174. 23377389

[30] Chiu H Wang T Chen P Hsu S Tsai Y Tsai T. Psoriasis in Taiwan: from epidemiology to new treatments. Dermatol Sin. 2018;36(3):115-123. 10.1016/j.dsi.2018.06.001

[31] Youn SW Tsai TF Theng C , et al MARCOPOLO Investigators. The MARCOPOLO study of ustekinumab utilization and efficacy in a real-world setting: treatment of patients with plaque psoriasis in Asia-Pacific countries. Ann Dermatol. 2016;28(2):222-231. 10.5021/ad.2016.28.2.222 27081271 PMC4828387

[32] Katoh N Kataoka Y Saeki H , et al Efficacy and safety of dupilumab in Japanese adults with moderate-to-severe atopic dermatitis: a subanalysis of three clinical trials. Br J Dermatol. 2020;183(1):39-51. doi:10.1111/bjd.18565 31564057 PMC7384164

[33] Zhao Y Wu L Lu Q , et al The efficacy and safety of dupilumab in Chinese patients with moderate-to-severe atopic dermatitis: a randomized, double-blind, placebo-controlled study. Br J Dermatol. 2022;186(4):633-641. doi:10.1111/bjd.20690 34358343 PMC9298048

[34] Zhou B Peng C Li L , et al Efficacy and safety of dupilumab in Chinese patients with atopic dermatitis: a real-world study. Front Med. 2022;9:838030. doi:10.3389/fmed.2022.838030 PMC8984471 35402441

[35] Vena GA Cassano N. Drug focus: adalimumab in the treatment of moderate to severe psoriasis. Biologics. 2007;1(2):93-103. 19707319 PMC2721299

[bibr36-12034754241260023] Harb H Chatila TA. Mechanisms of dupilumab. Clin Exp Allergy. 2020;50(1):5-14. doi:10.1111/cea.13491 31505066 PMC6930967

[37] Jeon C Sekhon S Yan D Afifi L Nakamura M Bhutani T. Monoclonal antibodies inhibiting IL-12, -23, and -17 for the treatment of psoriasis. Hum Vaccines Immunother. 2017;13(10):2247-2259. doi:10.1080/21645515.2017.1356498 PMC5647990 28825875

[38] Mineroff J Nguyen JK Jagdeo J. Racial and ethnic underrepresentation in dermatology clinical trials. J Am Acad Dermatol. 2023;89:293-300. doi:10.1016/j.jaad.2023.04.011 37062462

