# Supplementary material for: Response to Biologic Therapy in Skin of Colour Participants With Moderate-to-Severe Psoriasis and Atopic Dermatitis: A Systematic Review
Source: J Cutan Med Surg. 2024 Jun 7;28(5):468–72. doi: 10.1177/12034754241260023 (PMC11512488; doi:10.1177/12034754241260023)
Supplement: sj-docx-1-cms-10.1177_12034754241260023 – Supplemental material for Response to Biologic Therapy in Skin of Colour Participants With Moderate-to-Severe Psoriasis and Atopic Dermatitis: A Systematic Review [file sj-docx-1-cms-10.1177_12034754241260023.docx]

**Table S1:** **A Summary of Characteristics and Outcomes of Biologic Use in Skin of Colour Adult Participants with Psoriasis and Atopic Dermatitis.**

| Study Reference, country | Study Type (level of evidence) | Population (N) | Age (mean and SD or range) and sex | Condition | Intervention (N)  [route, dose, frequency] | Outcomes of Interest | Adverse Events (AE) | Discontinuations Due to Adverse events |
| --- | --- | --- | --- | --- | --- | --- | --- | --- |
| Asahina, 2023^1^  Japan | RCT (1b) | Japanese  N = 108 | Age: 51.6 ± 13  Male (82.4%) | Plaque Psoriasis | N = 17  Placebo [NR, NR, Q1W until week 16]🡪 Bimekizumab [NR, 320mg, Q4W until week 52]  N = 62 Bimekizumab [NR, 320mg, Q4W until week 52]  N = 29 Ustekinumab [IV; 45mg in <100kg, 90mg in >100kg on week: 0,4,16 then Q12W until week 52] | IGA 0/1 n(%)  Week 16: 0 (0%)  Week 52: 11 (64.7%)  PASI100 n(%)  Week 16: 0 (0%)  Week 52: 9 (52.9%)  PASI90 n(%)  Week 16: 1 (5.9%)  PASI75 n(%)  Week 16: 2 (11.8%)  Week 52: 13 (76.5%)  DLQI 0/1 n(%)  Week 16: 0 (0%)  Week 52: 12 (70.6%)  IGA 0/1 n(%)  Week 16: 51(82.3%)  Week 52: 46 (74.2%)  PASI100 n(%)  Week 16: 32 (51.6%)  Week 52: 31 (50%)  PASI90 n(%)  Week 16: 53 (85.5%)  Week 52: 50 (80.6%)  PASI75 n(%)  Week 16: 59 (95.2%)  Week 52: 53 (85.5%)  DLQI 0/1 n(%)  Week 16: 51 (82.3%)  Week 52: 43 (69.4%)  IGA 0/1 n(%)  Week 16: 14(48.3%)  Week 52: 13(44.8%)  PASI100 n(%)  Week 16: 4(13.8%)  Week 52: 8(27.6%)  PASI90 n(%)  Week 16: 15(51.7%)  Week 52: 14(48.3%)  PASI75 n(%)  Week 16: 21(72.4%)  Week 52: 20(69%)  DLQI 0/1 n(%)  Week 16: 14(48.3%)  Week 52: 17(58.6%) | Nasopharyngitis: 1  Nasopharyngitis: 8 Oral candidiasis: 6 Eczema: 2 Furuncle/ folliculitis: 2 Contact dermatitis: 2 Pharyngitis: 3 Tinea pedis: 3 Fungal infection: 11 Candida: 7 Tinea: 4 Abnormal liver function: 4  Nasopharyngitis: 2 Eczema: 1 | N = 0  N = 3    N = 0 |
| Cai, 2022^2^  China | RCT (1b) | Chinese  N = 262 | Age: 38 (18-75)  Male (72.5%) | Plaque Psoriasis  N = 262 | N = 131  HLX03 [Loading dose 80mg; 40mg Q2 🡪 IV, 40mg, Q2W until week 48]  N = 130 Adalimumab [Loading dose 80mg; 40mg Q2 🡪 IV; 40mg; Q2W until week 48] | PASI (% improvement from baseline)  Week 16: 83.5%  PASI75 n(%)  Week 16: 120 (92%)  Week 50: 126 (96%)  DLQI (mean change)  Week 16: -9.8  Week 50: -10.3  PASI (% improvement from baseline) Week 16: 82%  PASI75 n(%)  Week 16: 115 (88.7%)  Week 50: 121 (93%)  DLQI (mean change)  Week 16: -10.3  Week 50: -11 | URI: 37  Hyperuricaemia: 24  Abnormal liver function: 23  UTI: 16  Hyperlipidemia: 10  Nasopharyngitis: 10  URI: 34 Hyperuricaemia: 18 Abnormal liver function: 22 UTI: 12 Hyperlipidemia: 16 Nasopharyngitis: 13 | N = 1  N = 4 |
| Katoh, 2019^3^  Japan | RCT (1b) | Japanese  N = 223 | Trial 1:  Age: 40.01 (18-59)  Male (72.6%)  Trial 2:  Age: 34.205 (19 - 67)  Male (65.8%) | Atopic Dermatitis  N = 223 | N = 35  Placebo  [SC; 300mg; Q1W until week 16]  N = 36  Dupilumab [SC; 300mg; Q2W until week 16]    N = 35  Dupilumab [SC; 300mg; Q1W until week 16]  N = 54 TCS + Placebo  [SC; 300mg; Q1W until week 52]  N = 16  TCS + Dupilumab [SC; 300mg; Q2W until week 52]    N = 47  TCS + Dupilumab [SC; 300mg; Q1W until week 52] | IGA 0/1 n(%)  Week 16: 1(2.9%)  EASI75 n(%)  Week 16: 0(0%)  % EASI change from baseline  Week 16: 4.4    IGA 0/1 n(%)  Week 16: 7(19.4%)  EASI75 n(%)  Week 16: 9(25%)  % EASI change from baseline  Week 16: -61.5  IGA 0/1 n(%)  Week 16: 10(28.6%)  EASI75 n(%)  Week 16: 18(51.4%)  % EASI change from baseline  Week 16: -81  IGA 0/1 n(%)  Week 16: 2 (3.7%)  Week 52: 6(11.1%)  EASI75 n(%)  Week 16: 12(22.2%) Week 52: 13(24.1%)  % EASI change from baseline  Week 52: -64.9  IGA 0/1 n(%)  Week 16: 3 (18.8%) Week 52: 5 (31.3%)  EASI75 n(%)  Week 16: 10(62.5%) Week 52: 8 (50%)  % EASI change from baseline  Week 52: -85.5  IGA 0/1 n(%)  Week 16: 15(31.2%)  Week 52: 14 (29.8%)  EASI75 n(%)  Week 16: 30 (63.8%) Week 52: 33 (70.2%)  % EASI change from baseline  Week 52: -87.2 | UTI: 1  AD exacerbation: 1  AD exacerbation: 1  None  Pseudoarthrosis: 1 Cataracts: 1 Glaucoma: 1  None  Osteoarthritis: 1 | N  = 0  N = 0    N = 0  N = 3    N = 0    N = 2 |
| Lee, 2019^4^  USA, Netherlands, Taiwan, Australia, Canada, Germany, UK, Korea | RCT (1b) | Asian  N = 62 | Age: 39.6 ± 14.37  Male (79%) | Plaque Psoriasis  N = 62 | N = 23  Secukinumab [SC; 300mg; Q1W until week 3 🡪 SC; 300mg Q4W until week 48]  N = 39  Ustekinumab [SC; 45mg in <100kg, 90mg in >100kg on week: 0,4,16 then Q12W until week 52] | IGA 0/1 n(%)  Week 16: 17(73.9%) Week 52: 16(69.6%)  PASI100 n(%)  Week 16: 10(43.5%)  Week 52: 7(30.4%)  PASI90 n(%)  Week 16: 18(78.3%)  Week 52: 14(60.9%)  PASI75 n(%)  Week 16: 21(91.3%)  Week 52: 20(87%)  IGA 0/1 n(%) Week 16: 22(56.4%) Week 52: 18(43.6%)  PASI100 n(%)  Week 16:  4(10.3%)  Week 52: 5(12.8%)  PASI90 n(%)  Week 16: 14(35.9%)  Week 52: 13(33.3%)  PASI75 n(%)  Week 16: 26(66.7%)  Week 52: 22(56.4%) | URTI: 9  Nasopharyngitis: 7  Rhinorrhea: 4  Arthralgia: 3  Allergic conjunctivitis: 3  Diarrhea: 3  Eczema: 3  Headache: 3  Oropharyngeal Pain: 3  Vomiting: 3  Acne: 2  Contusion: 2  Cough: 2  Myalgia: 2  Pruritis: 2  Allergic rhinitis 2  Urticaria: 1  Ligament sprain: 1  Back pain: 1  Nausea: 2  Skin abrasion: 2  Tinea pedis: 2  URTI: 13 Nasopharyngitis: 3 Rhinorrhea: 3 Arthralgia: 5 Allergic conjunctivitis: 1 Diarrhea: 1 Eczema: 1 Headache: 3 Oropharyngeal Pain: 2 Acne: 3 Cough: 1 Pruritis: 3 Allergic rhinitis 3 Urticaria: 3 Ligament sprain: 1 Back pain: 2 Nausea: 2 Tinea pedis: 3 Psoriatic arthropathy: 2 Herpes: 3 | N = 1    N = 2 |
| Ohtsuki, 2014^5^  Japan | RCT (1b) | Japanese  N = 87 | Age: 50.1  Male (82.8%) | Plaque Psoriasis  N = 87 | N = 29  Placebo  [SC; 150mg; Q1W until week 4🡪 SC; 150mg; Q4W until week 12]  N = 29  Secukinumab  [SC; 150mg; Q1W until week 4🡪 SC; 150mg; Q4W until week 52]  N = 29  Secukinumab  [SC; 300mg; Q1W until week 4🡪 SC; 300mg; Q4W until week 52] | IGA 0/1 n(%)  Week 12: 1 (3.4%)  PASI100 n(%)  Week 12: 0 (0%)  PASI90 n(%)  Week 12: 0 (0%)  PASI75 n(%)  Week 12: 2 (6.9%)  DLQI 0/1 n(%)  Week 12: 7(24.1%)  IGA 0/1 n(%) Week 12: 16 (55.2%) Week 16: 16 (55.2%) Week 52: 10 (34.5%)  PASI100 n(%)  Week 12: 3 (10.2%)  Week 16: 2 (6.9%)  Week 52: 10 (34.5%)  PASI90 n(%)  Week 12: 16 (55.2%)  Week 16: 20 (69%)  Week 52: 12 (41.4%)  PASI75 n(%)  Week 12: 25 (86.2%)  Week 16: 25 (86.2%)  Week 52: 22 (75.9%)  DLQI 0/1 n(%)  Week 12: 19 (65.5%)  Week 52: 19 (65.5%)  IGA 0/1 n(%)  Week 12: 16 (55.2%)  Week 16: 18 (62.1%)  Week 52: 18 (62.1%)  PASI100 n(%)  Week 12: 8 (27.6%)  Week 16: 12 (41.4%)  Week 52: 18 (62.1%)  PASI90 n(%)  Week 12: 18 (62.1%)  Week 16: 21 (72.4%)  Week 52: 20  (69%)  PASI75 n(%)  Week 12: 24 (82.8%)  Week 16: 24 (82.8%)  Week 52: 25 (86.2%)  DLQI 0/1 n(%)  Week 12: 20 (95.2%)  Week 52: 21 (72.4%) | Nasopharyngitis: 5  Pruritis: 2    Nasopharyngitis: 20  Folliculitis/ Furuncle: 2  Pharyngitis: 4  Tinea Pedis: 2  Pruritis: 1  Eczema: 4  Urticaria: 3    Nasopharyngitis: 8 Folliculitis/ Furuncle: 2 Tinea Pedis: 2 Pruritis: 1 Eczema: 3 Urticaria: 2 | N = 1    N = 4  N = 0 |
| Okubo, 2018^6^  Japan | RCT (1b) | Japanese  N = 13 | Age: 49.15 (29-69)  Male (69%) | Erythro- dermic  psoriasis  N = 8  Generalized  Pustular Psoriasis  N = 5 | N = 8  Ixekizumab [Loading dose SC; 160mg 🡪 SC; 80mg; Q2W until week 12 🡪 SC; 80mg; Q4W until week 244]    N = 5  Ixekizumab [Loading dose SC; 160mg 🡪 SC; 80mg; Q2W until week 12 🡪 SC; 80mg; Q4W until week 244] | PASI (mean)  Week 52: 3.0  Week 244: 5.0  DLQI (mean and SD)  Week 12: 1.8 ± 3.0 Week 52: 1.9 ± 1.7  Week 244: 2.1 ± 2.5  PASI (mean)  Week 52: 1.8  Week 244: 1.6  DLQI (mean and SD)  Week 12: 4.2 ± 6.6  Week 52: 3.8 ± 4.4  Week 244: 3.6 ± 4.8 | Infection unspecified: 5  URTI: 2  Eczema: 1  Abnormal liver function: 2  Infection unspecified: 5 URTI: 2 Contact dermatitis: 1 Allergic rhinitis: 1 Eczema: 1 | N = 2  N = 0 |
| Tsai, 2011^7^  Korea and Taiwan | RCT (1b) | Korean  N = 61  Taiwanese  N = 30  Chinese  N = 30 | Age: 40.6 ± 11.4  Male (85.1%) | Plaque Psoriasis  N = 121 | N = 60  Placebo  [SC; 45mg; on week: 0, 4, 16 until week 28]  N = 61  Ustekinumab  [SC; 45mg; on week: 0, 4, 16 until week 28] | PASI100 n(%)  Week 12: 0(0%)  Week 28: 9(16.7%)  PASI90 n(%)  Week 12: 1(1.7%)  Week 28: 25(46.3%)  PASI75 n(%)  Week 12: 3(5%)  Week 28: 40(72.4%)  PASI50 n(%)  Week 12: 8(13.3%)  Week 28: 47(873%)  PASI (mean CFB)  Week 12: 3.1 ± 43.0  Week 28: 78.3 ± 25.8  DLQI (mean CFB)  Week 12: -0.5 ± 6.5 Week 28: -10.0 ± 6.4  PASI100 n(%)  Week 12: 5(8.2%)  Week 28: 12(20.7%)  PASI90 n(%)  Week 12: 30(49.2%)  Week 28: 35(60.3%)  PASI75 n(%)  Week 12: 41(67.2%)  Week 28: 42(72.4%)  PASI50 n(%)  Week 12: 51(83.6%)  Week 28: 49(84.5%)  PASI (mean CFB)  Week 12: 78.5 ± 27.2  Week 28: 79.5 ± 30  DLQI (mean CFB)  Week 12: -11.2 ± 7.1 Week 28: -10.8 ± 7.5 | URTI: 12  Hyperglycemia: 8  Nasopharyngitis: 13  Pruritis: 9  Cough: 4  Eosinophilia: 5  Anemia: 2  Injection site reaction: 1  Eczema: 3  Abnormal liver function: 5    URTI: 9  Hyperglycemia: 8  Nasopharyngitis: 6  Pruritis: 18  Cough: 5  Eosinophilia: 3  Anemia: 4  Injection site reaction: 4  Eczema: 2  Abnormal liver function: 6  Psoriatic arthropathy: 3 | N = 1    N = 3 |
| Wu, 2017^8^  Taiwan | RCT (1b) | Taiwanese  N = 51 | Age: 39.35  Male (80%) | Plaque Psoriasis  N = 51 | N = 15  Placebo  [SC; 150mg; Q1W until week 4🡪 SC; 150mg; Q4W until week 12]  N = 20 Secukinumab  [SC; 150mg; Q1W until week 4🡪 SC; 150mg; Q4W until week 52]    N = 16  Secukinumab  [SC; 300mg; Q1W until week 4🡪 SC; 300mg; Q4W until week 52] | IGA 0/1 n(%)  Week 12: 0 (0%)  PASI100 n(%)  Week 12: 0 (0%)  PASI90 n(%)  Week 12: 0 (0%)  PASI75 n(%)  Week 12: 0 (0%)  IGA 0/1 n(%) Week 12: 13(65%) Week 16: 7(35%) Week 52: 7(35%)  PASI100 n(%)  Week 12: 3(15%)  Week 52: 3(15%)  PASI90 n(%)  Week 12: 9(45%)  Week 52: 7(35%)  PASI75 n(%)  Week 12: 14(70%)  Week 52: 12(60%)  IGA 0/1 n(%)  Week 12: 11 (68.9%)  Week 16: 8(50%) Week 52: 8(50%)  PASI100 n(%)  Week 12: 5(31.3%)  Week 52: 7(43.%)  PASI90 n(%)  Week 12: 11(68.8%)  Week 52: 10(62.5%)  PASI75 n(%)  Week 12: 14(87.5%)  Week 52: 11(68.8%) | Headache: 2  Cough: 1  URTI: 2  furuncle/ folliculitis: 1  Periodontitis: 2  Hyperlipidemia: 4  Headache: 4 Cough: 1 URTI: 15 Furuncle/ folliculitis: 3 Periodontitis: 4 Diarrhea: 3 Pruritis: 8 Eczema: 6 Urticaria: 4 Hyperlipidemia: 3    Headache: 3 Cough: 4 URTI: 12 Furuncle/ folliculitis: 3 Periodontitis: 4 Diarrhea: 1 Pruritis: 10 Eczema: 3 Urticaria: 3 Hyperlipidemia: 4 | N = 0  N = 0    N = 0 |
| Yu, 2022^9^  China | RCT (1b) | Chinese  N = 367 | Age: 39 (18-70)  Male (80.9%) | Plaque Psoriasis  N = 367 | N = 184  SCT630  [Loading dose SC 80mg; 40mg Q2 🡪 SC; 40mg; Q2W until week 16]  N = 183 Adalimumab [Loading dose SC 80mg; 40mg Q2 🡪 SC; 40mg; Q2W until week 16] | PASI (% improvement from baseline)  Week 16: 85.07%  DLQI (mean change)  Week 16: -11.25  PASI (% improvement from baseline)  Week 16: 84.82%  DLQI (mean change)   Week 16: -12.5 | Injection site reaction: 0  Respiratory Tract Infection): 9  TB: 1  Infection unspecified: 3  Injection site reaction: 3 Respiratory Tract Infection): 3 TB: 2 Infection unspecified: 2 | N = 7  N = 3 |
| Zhao 2021^10^  China | RCT (1b) | Chinese  N = 165 | Age: 30.6  Male (71.5%) | Atopic Dermatitis  N = 165 | N = 83  Placebo  [Loading dose SC 600mg 🡪 SC; 300mg; Q2W until week 16]    N = 82  Dupilumab  [Loading dose SC 600mg 🡪 SC; 300mg; Q2W until week 16] | IGA 0/1 n(%)  Week 16: 4 (4.8%)  EASI90 n(%)  Week 16: 5 (6.0%)  EASI75 n(%)  Week 16: 12 (14.5%)  EASI50 n(%)  Week 16: 24 (28.9%)  BSA (%CFB mean and SE)  Week 16: −19.3±2.56  DLQI (% CFB mean and SE)  Week 16: −10.3±0.63  IGA 0/1 n(%)  Week 16: 22(26.8%)  EASI90 n(%)  Week 16: 33 (40.2%)  EASI75 n(%)  Week 16: 47 (57.3%)  EASI50 n(%)  Week 16: 58 (70.7%)  BSA (%CFB mean and SE)  Week 16: −37.8±2.13  DLQI (%CFB mean and SE) Week 16: −5.06±0.71 | Infection unspecified: 21  URTI: 6  Conjunctivitis: 4  Eye disorder: 4  Skin/ subcutaneous tissue disorders: 40  Injection site reaction: 2  Proteinuria: 7  Increased blood uric acid levels: 5    Infection unspecified: 28 URTI: 9 Conjunctivitis: 7 Eye disorder: 9 Skin/ subcutaneous disorder: 26 Injection site reaction: 7 Proteinuria: 7 Increased blood uric acid levels: 1 | N = 4  N = 2 |
| Zhu 2013^11^  China | RCT (1b) | Chinese  N = 322 | Age: 39.7±12.3  Male (77%) | Plaque Psoriasis  N = 322 | N = 162 Placebo [SC; 45mg; on week: 0 and 4] 🡪 Ustekinumab [SC; 45mg; on week: 12, 16]    N = 160 Ustekinumab  [SC; 45mg; 45mg; on week: 0, 4, 16] | PASI100  Week 12: 1(0.6%)  Week 28: 63(38.9%)  PASI90  Week 12: 5(3%)  Week 28: 112(69.1%)  PASI75  Week 12: 18(11.1%)  Week 28: 135(83.3%)  PASI50  Week 12: 32 (19.8%)  Week 28: 145 (89.5%)  PASI (mean CFB)  Week 12: 12.9±45.3  Week 28: 92.3±13  DLQI (mean CFB)  Week 12: -1.9±6.6  Week 28: -9.9±7.5  PASI100  Week 12: 38(23.8%)  Week 28: 62(38.8%)  PASI90  Week 12: 107(66.9%)  Week 28: 123(76.9%)  PASI75  Week 12: 132(82.5%)  Week 28: 140(87.5%)  PASI50  Week 12: 146 (91.3%)  Week 28: 151(94.4%)  PASI (mean CFB):  Week 12: 86.8±19.7  Week 28: 93±12.2  DLQI (mean CFB)  Week 12: -9.3±7.2  Week 28: -10.7±7.7 | Nasopharyngitis: 26  URTI: 16  Cough: 9  Rhinorrhea: 7  Pruritis: 5  Pharyngitis: 5  Leukopenia: 3  Oropharyngeal pain: 4  Pyrexia: 6  Arthralgia: 4  Infection unspecified: 65  Abnormal liver function: 9  Nasopharyngitis: 28 URTI: 20 Cough: 11 Rhinorrhea: 4 Pruritus: 8 Pharyngitis: 4 Leukopenia: 5 Oropharyngeal pain: 6 Pyrexia: 1 Arthralgia: 8 Infection unspecified: 69 Abnormal liver function: 15 | N = 5  N = 2 |

**Abbreviations:** AD denotes Atopic Dermatitis, BSA denotes Body surface area, CFB denotes change from baseline, DLQI denotes Dermatology Life Quality Index, EASI denotes Eczema Area and Severity Index, HLX03 denotes adalimumab biosimilar, IGA denotes Investigators Global Assessment, IV denotes intravenous, N denotes number of participants/ population sample, NR denotes not recorded, PASI denotes Psoriasis Area and Severity Index, Q2 denotes day 2, Q1W denotes every 1 week, Q2W denotes every 2 weeks, Q4W denotes every 4 weeks, Q12W denotes every 12 weeks, RCT denotes Randomized Controlled Trial, SC denotes subcutaneous, SCT630 denotes adalimumab biosimilar, SD (±) denotes standard deviation, SE (±) denotes standard error, TB denotes tuberculosis, TCS denotes topical corticosteroids, URI denotes Upper Respiratory Infection, URTI denotes Upper Respiratory Tract Infection, UTI denotes Urinary Tract Infection.

**References:**

1. Asahina, A., Okubo, Y., Morita, A., Tada, Y., Igarashi, A., Langley, R. G., Deherder, D., Matano, M., Vanvoorden, V., Wang, M., Ohtsuki, M., & Nakagawa, H. (2023). Bimekizumab Efficacy and Safety in Japanese Patients with Plaque Psoriasis in BE VIVID: A Phase 3, Ustekinumab and Placebo-Controlled Study. *Dermatology and therapy*, *13*(3), 751–768. <https://doi.org/10.1007/s13555-022-00883-y>
2. Cai, L., Li, L., Cheng, H., Ding, Y., Biao, Z., Zhang, S., Geng, S., Liu, Q., Fang, H., Song, Z., Lu, Y., Li, S., Guo, Q., Tao, J., He, L., Gu, J., Yang, Q., Han, X., Gao, X., Deng, D., … Zhang, J. (2022). Efficacy and Safety of HLX03, an Adalimumab Biosimilar, in Patients with Moderate-to-Severe Plaque Psoriasis: A Randomized, Double-Blind, Phase III Study. *Advances in therapy*, *39*(1), 583–597. <https://doi.org/10.1007/s12325-021-01899-0>
3. Katoh, N., Kataoka, Y., Saeki, H., Hide, M., Kabashima, K., Etoh, T., Igarashi, A., Imafuku, S., Kawashima, M., Ohtsuki, M., Fujita, H., Arima, K., Takagi, H., Chen, Z., Shumel, B., & Ardeleanu, M. (2020). Efficacy and safety of dupilumab in Japanese adults with moderate-to-severe atopic dermatitis: a subanalysis of three clinical trials. *The British journal of dermatology*, *183*(1), 39–51. <https://doi.org/10.1111/bjd.18565>
4. Lee, M. G., Huang, Y. H., Lee, J. H., Lee, S. C., Kim, T. G., Aw, D. C., Bao, W., Dee, C. M. A., Guana, A., & Tsai, T. F. (2019). Secukinumab demonstrates superior efficacy and a faster response in clearing skin in Asian subjects with moderate to severe plaque psoriasis compared with ustekinumab: Subgroup analysis from the CLEAR study. *The Journal of dermatology*, *46*(9), 752–758. <https://doi.org/10.1111/1346-8138.15004>
5. Ohtsuki, M., Morita, A., Abe, M., Takahashi, H., Seko, N., Karpov, A., Shima, T., Papavassilis, C., Nakagawa, H., & ERASURE Study Japanese subgroup (2014). Secukinumab efficacy and safety in Japanese patients with moderate-to-severe plaque psoriasis: subanalysis from ERASURE, a randomized, placebo-controlled, phase 3 study. *The Journal of dermatology*, *41*(12), 1039–1046. <https://doi.org/10.1111/1346-8138.12668>
6. Okubo, Y., Mabuchi, T., Iwatsuki, K., Elmaraghy, H., Torisu-Itakura, H., Morisaki, Y., & Nakajo, K. (2019). Long-term efficacy and safety of ixekizumab in Japanese patients with erythrodermic or generalized pustular psoriasis: subgroup analyses of an open-label, phase 3 study (UNCOVER-J). *Journal of the European Academy of Dermatology and Venereology : JEADV*, *33*(2), 325–332. <https://doi.org/10.1111/jdv.15287>
7. Tsai, T. F., Ho, J. C., Song, M., Szapary, P., Guzzo, C., Shen, Y. K., Li, S., Kim, K. J., Kim, T. Y., Choi, J. H., Youn, J. I., & PEARL Investigators (2011). Efficacy and safety of ustekinumab for the treatment of moderate-to-severe psoriasis: a phase III, randomized, placebo-controlled trial in Taiwanese and Korean patients (PEARL). *Journal of dermatological science*, *63*(3), 154–163. <https://doi.org/10.1016/j.jdermsci.2011.05.005>
8. Wu, N. L., Hsu, C. J., Sun, F. J., & Tsai, T. F. (2017). Efficacy and safety of secukinumab in Taiwanese patients with moderate to severe plaque psoriasis: Subanalysis from ERASURE phase III study. *The Journal of dermatology*, *44*(10), 1129–1137. <https://doi.org/10.1111/1346-8138.13900>
9. Yu, C., Zhang, F., Ding, Y., Li, Y., Zhao, Y., Gu, J., Guo, S., Pan, W., Jin, H., Sun, Q., Kang, X., Yang, Q., Jiang, X., Song, Z., Lu, Q., Pang, X., Kuang, Y., Deng, D., Li, Y., Zhang, C., … Wang, G. (2022). A randomized, double-blind phase III study to demonstrate the clinical similarity of biosimilar SCT630 to reference adalimumab in Chinese patients with moderate to severe plaque psoriasis. *International immunopharmacology*, *112*, 109248. <https://doi.org/10.1016/j.intimp.2022.109248>
10. Zhao, Y., Wu, L., Lu, Q., Gao, X., Zhu, X., Yao, X., Li, L., Li, W., Ding, Y., Song, Z., Liu, L., Dang, N., Zhang, C., Liu, X., Gu, J., Wang, J., Geng, S., Liu, Q., Guo, Y., Dong, L., … Zhang, J. (2022). The efficacy and safety of dupilumab in Chinese patients with moderate-to-severe atopic dermatitis: a randomized, double-blind, placebo-controlled study. *The British journal of dermatology*, *186*(4), 633–641. <https://doi.org/10.1111/bjd.20690>
11. Zhu, X., Zheng, M., Song, M., Shen, Y. K., Chan, D., Szapary, P. O., Wang, B., & LOTUS Investigators (2013). Efficacy and safety of ustekinumab in Chinese patients with moderate to severe plaque-type psoriasis: results from a phase 3 clinical trial (LOTUS). *Journal of drugs in dermatology : JDD*, *12*(2), 166–174.
