# Supplementary material for: Response to Biologic Therapy in Skin of Colour Participants With Moderate-to-Severe Psoriasis and Atopic Dermatitis: A Systematic Review
Source: J Cutan Med Surg. 2024 Jun 7;28(5):468–72. doi: 10.1177/12034754241260023 (PMC11512488; doi:10.1177/12034754241260023)
Supplement: sj-pdf-3-cms-10.1177_12034754241260023 – Supplemental material for Response to Biologic Therapy in Skin of Colour Participants With Moderate-to-Severe Psoriasis and Atopic Dermatitis: A Systematic Review [file sj-pdf-3-cms-10.1177_12034754241260023.pdf]

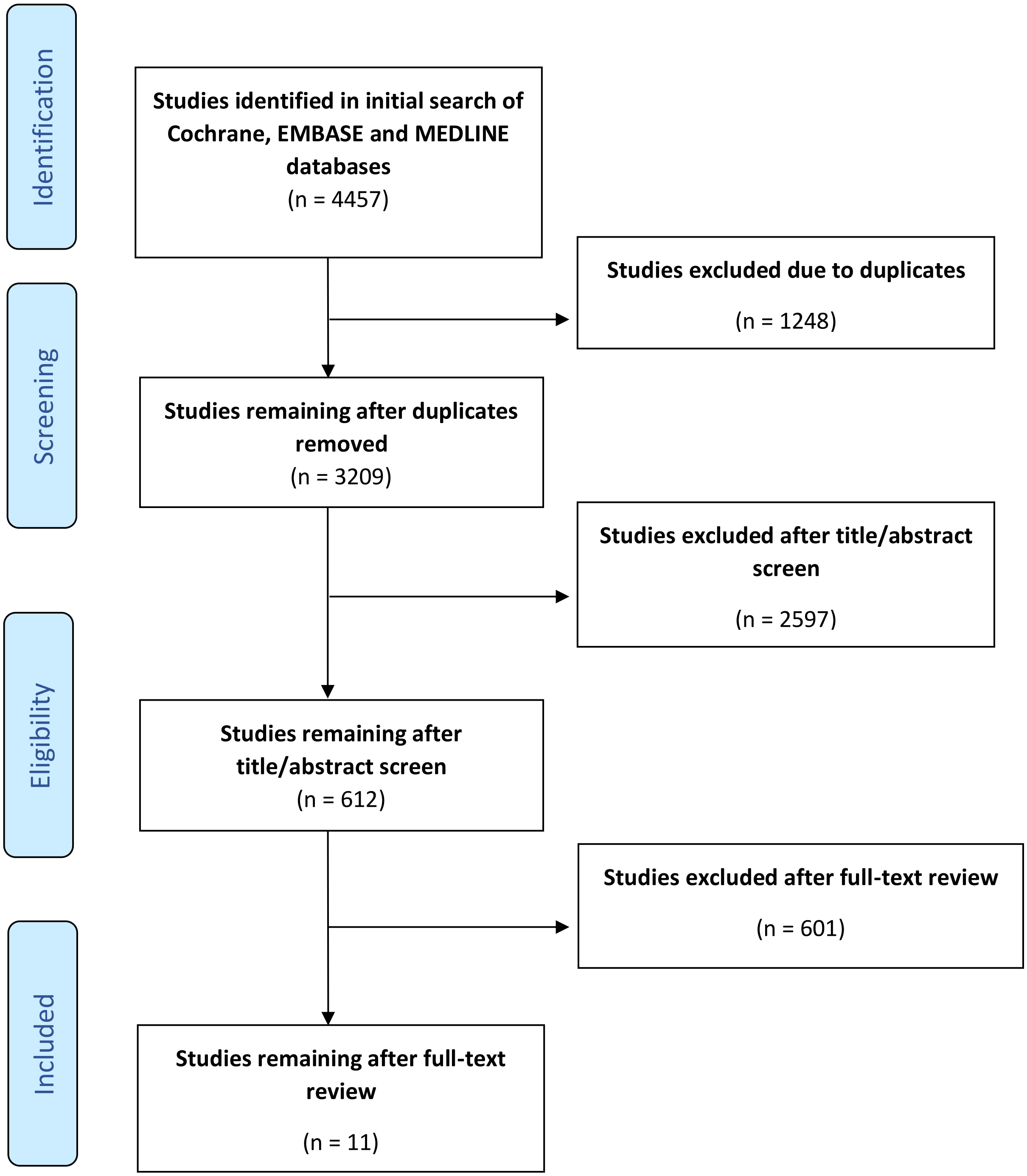

The criteria for study inclusion were: (i) conducted a phase 3 trial, (ii) included adult participants, (iii) documented participants with moderate to severe atopic dermatitis or psoriasis, (iv) used biologics as an intervention, and (v) documented separate results and outcomes of treatment in non-white participants

Figure S2. Flow diagram of literature screening using the Preferred Reporting Items for Systematic Reviews and Meta-Analyses (PRSMA) guidelines. Figure adapted from http://prisma-statement.org.
