# Supplementary material for: Response to Biologic Therapy in Skin of Colour Participants With Moderate-to-Severe Psoriasis and Atopic Dermatitis: A Systematic Review
Source: J Cutan Med Surg. 2024 Jun 7;28(5):468–72. doi: 10.1177/12034754241260023 (PMC11512488; doi:10.1177/12034754241260023)
Supplement: sj-pdf-4-cms-10.1177_12034754241260023 – Supplemental material for Response to Biologic Therapy in Skin of Colour Participants With Moderate-to-Severe Psoriasis and Atopic Dermatitis: A Systematic Review [file sj-pdf-4-cms-10.1177_12034754241260023.pdf]

Intention-to-treat

Unique ID	Study ID	Experimental	Comparator	Outcome	Weight	D1	D2	D3	D4	D5	Overall
1	Asahina et al. 2023	NA	NA	NA	1	+	+	+	+	+	+
2	Cai et al. 2022	NA	NA	NA	1	+	+	+	+	+	+
3	Katoh et al. 2019	NA	NA	NA	1	+	+	+	+	+	+
4	Lee et al. 2019	NA	NA	NA	1	+	+	+	+	+	+
5	Ohtsuki et al. 2014	NA	NA	NA	1	+	+	+	+	+	+
6	Okubo et al. 2018	NA	NA	NA	1	!	+	+	-	+	-
7	Tsai et al. 2011	NA	NA	NA	1	+	+	+	+	+	+
8	Wu et al. 2017	NA	NA	NA	1	+	+	+	+	+	+
9	Yu et al. 2022	NA	NA	NA	1	+	+	+	+	+	+
10	Zhao et al. 2021	NA	NA	NA	1	+	+	+	+	+	+
11	Zhu et al. 2013	NA	NA	NA	1	+	+	+	+	+	+

+ Low risk
! Some concerns
- High risk

D1 Randomisation process
D2 Deviations from the intended interventions
D3 Missing outcome data
D4 Measurement of the outcome
D5 Selection of the reported result
