# Supplementary material for: Response to Biologic Therapy in Skin of Colour Participants With Moderate-to-Severe Psoriasis and Atopic Dermatitis: A Systematic Review
Source: J Cutan Med Surg. 2024 Jun 7;28(5):468–72. doi: 10.1177/12034754241260023 (PMC11512488; doi:10.1177/12034754241260023)
Supplement: sj-pdf-5-cms-10.1177_12034754241260023 – Supplemental material for Response to Biologic Therapy in Skin of Colour Participants With Moderate-to-Severe Psoriasis and Atopic Dermatitis: A Systematic Review [file sj-pdf-5-cms-10.1177_12034754241260023.pdf]

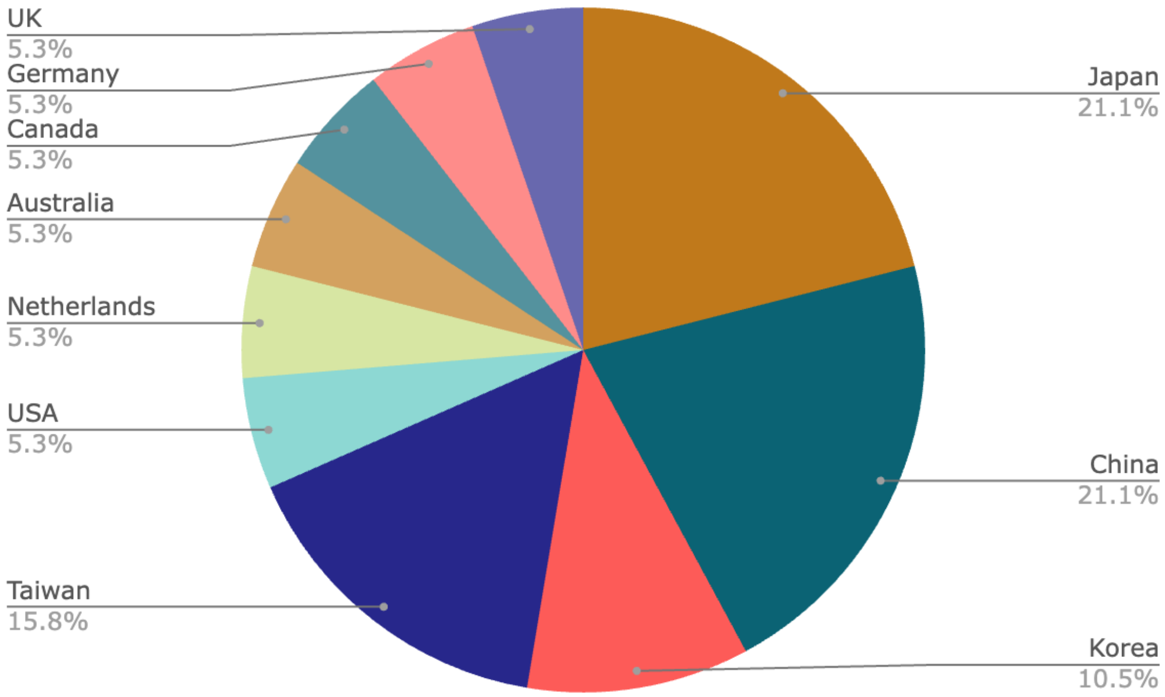

Figure S4. Country of phase 3 trial conduct. Countries included Japan (n = 4), China (n = 4), Korea (n = 2), Taiwan (n = 3), USA (n = 1), Netherlands (n = 1), Australia (n = 1), Canada (n = 1), Germany (n = 1), UK (n = 1).
