# Supplementary material for: Response to Biologic Therapy in Skin of Colour Participants With Moderate-to-Severe Psoriasis and Atopic Dermatitis: A Systematic Review
Source: J Cutan Med Surg. 2024 Jun 7;28(5):468–72. doi: 10.1177/12034754241260023 (PMC11512488; doi:10.1177/12034754241260023)
Supplement: sj-pdf-7-cms-10.1177_12034754241260023 – Supplemental material for Response to Biologic Therapy in Skin of Colour Participants With Moderate-to-Severe Psoriasis and Atopic Dermatitis: A Systematic Review [file sj-pdf-7-cms-10.1177_12034754241260023.pdf]

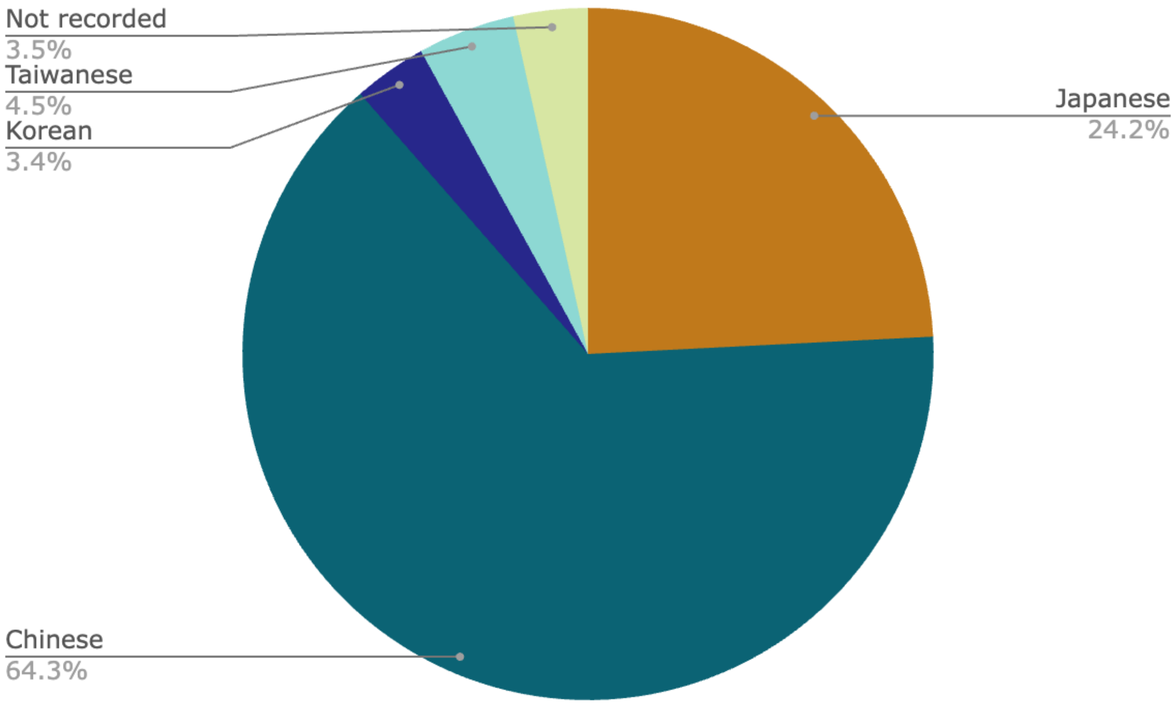

Figure S6. Documented skin of colour demographic. Categorized by ethnicity: Chinese (*n* = 1146), Japanese (*n* = 431), Taiwanese (*n* = 81), Korean (*n* = 61), not recorded (*n* = 62).
